# Nanoantioxidants: Recent Trends in Antioxidant Delivery Applications

**DOI:** 10.3390/antiox9010024

**Published:** 2019-12-26

**Authors:** Ibrahim Khalil, Wageeh A. Yehye, Alaitz Etxabide Etxeberria, Abeer A. Alhadi, Seyedehsara Masoomi Dezfooli, Nurhidayatullaili Binti Muhd Julkapli, Wan Jefrey Basirun, Ali Seyfoddin

**Affiliations:** 1 Nanotechnology and Catalysis Research Centre (NANOCAT), Institute for Advanced Studies, University of Malaya, Kuala Lumpur 50603, Malaysia; ikhalilcu@gmail.com (I.K.); nurhidayatullaili@um.edu.my (N.B.M.J.); jeff@um.edu.my (W.J.B.); 2 Drug Delivery Research Group, School of Science, Faculty of Health and Environmental Sciences, Auckland University of Technology, Auckland 0627, New Zealand; alaitz.etxabide@gmail.com (A.E.E.); sara.masoomi.dezfooli@aut.ac.nz (S.M.D.); 3 Department of Chemistry, Faculty of Science, University of Malaya, Kuala Lumpur 50603, Malaysia; abeer@um.edu.my; 4 Drug Design and Development Research Group, Department of Chemistry, Faculty of Science, University of Malaya, Kuala Lumpur 50603, Malaysia; 5 School of Interprofessional Health Studies, Faculty of Health and Environmental Sciences, Auckland University of Technology, Auckland 1142, New Zealand

**Keywords:** antioxidant, nanoparticles, nanoantioxidant, antioxidant delivery, nanocarrier, nanoencapsulation

## Abstract

Antioxidants interact with free radicals, terminating the adverse chain reactions and converting them to harmless products. Antioxidants thus minimize the oxidative stress and play a crucial role in the treatment of free radicals-induced diseases. However, the effectiveness of natural and/or synthetic antioxidants is limited due to their poor absorption, difficulties to cross the cell membranes, and degradation during delivery, hence contributing to their limited bioavailability. To address these issues, antioxidants covalently linked with nanoparticles, entrapped in nanogel, hollow particles, or encapsulated into nanoparticles of diverse origin have been used to provide better stability, gradual and sustained release, biocompatibility, and targeted delivery of the antioxidants with superior antioxidant profiles. This review aims to critically evaluate the recent scientific evaluations of nanoparticles as the antioxidant delivery vehicles, as well as their contribution in efficient and enhanced antioxidant activities.

## 1. Introduction

Antioxidants are molecules that can interact with free radicals in a safe manner, terminate the chain reaction, and convert them to a harmless molecule by donating an electron [1]. In the current decade, antioxidants have drawn attention due to their potential to minimize oxidative stress, which is defined as the pathophysiological response, created due to the imbalance between the production of oxidants and the endogenous antioxidants to counteract. Hence, there is a net increase of the reactive oxygen species (ROS) including superoxide anions, hydroxyl radicals (HO•), hydrogen peroxide (H_2_O_2_), singlet oxygen (^1^O_2_), and reactive nitrogen species (RNS) (e.g., peroxynitrite, peroxyl radicals) [2]. ROS and RNS are generally induced by both endogenous routes include ROS generating enzymes, such as nitric oxide synthase, xanthine oxidase, and metabolism (e.g., secretion by macrophages, as well as by-product formation by the electron transport chain), and external sources including environmental stress (e.g., ionizing radiation, redox, and heavy metals, excess UV-radiation). ROS and RNS may react with and cause damage to cell membranes, membranes of subcellular organelles, proteins, lipids, and DNA, and thus impair the normal functioning of the cells, subsequently leading to mutations, apoptosis, and failure within these systems [3,4,5]. Thus, at a systematic level, oxidative stress has been shown to play a role in the development and acceleration of many diseases, including diabetes, cardiovascular diseases, Alzheimer’s and Parkinson’s disease, acute renal failure, acute lung injury, radiation injury, cancer, arthritis, and even aging [2,3,6]. Almost all organisms have in-built endogenous antioxidant defense and repair systems for protection against oxidative damage, and very frequently these systems are inadequate to completely prevent the damage [7]. Therefore, the use of antioxidant supplements is recommended to reduce the oxidative damage to the human body. Antioxidants generally exert their activity mainly by two basic ways, either by preventing the formation of ROS/RNS or scavenging/neutralizing ROS/RNS. In some cases, antioxidants, specifically enzymatic compounds, also show antioxidant activity by decomposing ROS/RNS into less harmful or neutral products [3,8].

Many different chemical compounds have been evaluated for their antioxidant properties. They may be either endogenous (e.g., glutathione and uric acid) or exogenous, based on the source of origin, but the majority of antioxidants come from our diet [9]. These may be naturally derived, synthetic or inorganic material at nanoscale level, or, the most interesting and recently explored, nano-encapsulated antioxidant molecules. Natural antioxidants have the ability to potentially modulate the oxidative stress. Several fruits, vegetables, and fruit by-products have been screened for antioxidant contents such as vitamins (ascorbic acid), carotenoids (lutein), polyphenols (3,6-dihydroxyflavone), and metabolic sensitizers (methyl selenocysteine), which have been shown to scavenge the excess free radicals from the human body [10,11,12,13]. Moreover, antioxidants such as gallic acid (GA) derived from natural resources (dried rose flower extracts—Rosa rugosa), as well as bioactive compounds derived from different sources, have also exhibited potential antioxidant capacity, ability to scavenge active oxygen species and electrophiles, inhibition of nitrosation reactions, and in the decrement of lipid peroxidation level [14,15]. However, natural antioxidants are prone to degradation and their bioavailability is limited by the low absorption and the degradation during delivery [16]. Some synthetic compounds, such as butylated hydroxyanisole (BHA) and butylated hydroxytoluene (BHT), BHT analogs, and GA esters have also been used as antioxidants [10,17,18], which have shown negative heath influences, hence their applications have been restricted and replaced by the naturally occurring dietary antioxidants [13].

Advancement in nanotechnology has revealed several nanoparticles either from inorganic [19,20] or biological origins, such as melanin nanoparticles [21] as potent antioxidants by themselves. Novel metal nanoparticles (Au, Ag, Pt) and transition metal oxide (CuO, NiO) are the commonly used and tested for their antioxidant activity [19,20,22,23,24]. Moreover, nanocomposites either in single or bi-metallic combination, synthesized via chemical or green techniques using different phytochemicals (leaf extracts), were also evaluated for antioxidant activity [23,25,26]. However, the antioxidant properties of these nanoparticles depends on their nature, chemical composition, surface charge, particle size, surface to volume ratio, and surface coating [27]. Nanoparticles offer several advantages over traditional antioxidant delivery methods, which include environmental protection of bioactive components, increased bioavailability, and targeted delivery of antioxidants, as well as controlled release at the site of action [16]. However, achieving high specificity against ROS is the main hurdle to these antioxidants, which consequently may fail to prevent oxidative damage completely. In addition, oxidative stress, immune cell activation, mitochondrial respiration, and genotoxicity are the major challenging issues for in vivo applications of these exogenous nanomaterials, which may give rise to potential deleterious health effects [28].

Engineered nanostructured particles have recently been considered as an innovative strategy to provide novel antioxidants with enhanced characteristics. Nanoparticles functionalized with natural antioxidants or antioxidant enzymes where nanoparticles act as the carrier or antioxidant delivery vehicle were found to be efficient in enhancing the antioxidant activity and providing targeted delivery of certain antioxidants that show poor permeation across cell membranes and cell internalization [3,29]. Sharpe et al., who used the term nanoantioxidant for the first time, classified the nanoparticle antioxidants into two categories: (i) inorganic nanoparticles with intrinsic antioxidant properties, and (ii) functionalized and composite inorganic nanoparticle antioxidant. The latter one, consequently, was subdivided into three classes based on the mode of integration of the antioxidants, such as antioxidant functionalized nanoparticles, nanoencapsulated antioxidant, and magnetically responsive antioxidant nanocarrier [3]. Surface antioxidants-functionalized nanomaterials have demonstrated increased antioxidant activity and bioavailability [27,30]. Biodegradable nanoparticles have been used to improve the bioavailability of natural antioxidants, reduce the leaching and volatility, or to mimic potential enzyme functions in the biological systems [31]. Moreover, inorganic nanoparticles are thermally stable and chemically inert, which facilitate exploiting the potential, as well as the immobilization of natural antioxidants. Furthermore, nanoparticles conjugated natural antioxidants facilitate chemical stability of the antioxidants in physiological conditions, deliver the product in intact molecular form in a wider concentration range, and most importantly offer slow and continuous release [32].

There are many studies on natural, synthetic, and nanoparticle antioxidants and their potentials in diverse applications, including gene delivery [33], for theranostics in neurodegenerative [34,35] and cardiovascular diseases [36], biomedical applications [37,38], and therapy for different environmental pollutants-induced toxicities [39]. However, there is no study yet, as per our knowledge, on the range of nanoparticles and the way of antioxidant integration to have a complete up-to-date picture about this field in a broader context. Therefore, in this review we focus on the different strategies for the functionalization of nanoparticles with antioxidants or compounds possessing antioxidant properties for the efficient and targeted delivery with sustained release properties. In addition, the commonly used techniques for the integration of the antioxidants, including nanostructures surface loading, encapsulation, entrapped in the nanogel or in the core and/or core-shell spaces of hollow nanospheres, along with the consequent effects on the antioxidant properties and their targeted delivery, have been discussed herewith. Hence, in this review, nanoantioxidants were broadly categorized into: nanoparticle functionalized, nanogel entrapped, hollow nanosphere tagged and nanoparticle encapsulated antioxidants and the detail discussion in respect to properties, stability, potency, and most importantly, delivery strategies have been outlined.

## 2. Antioxidant Functionalized Nanoparticles

### 2.1. SiO_2_ Nanoantioxidant

**Figure 1:**
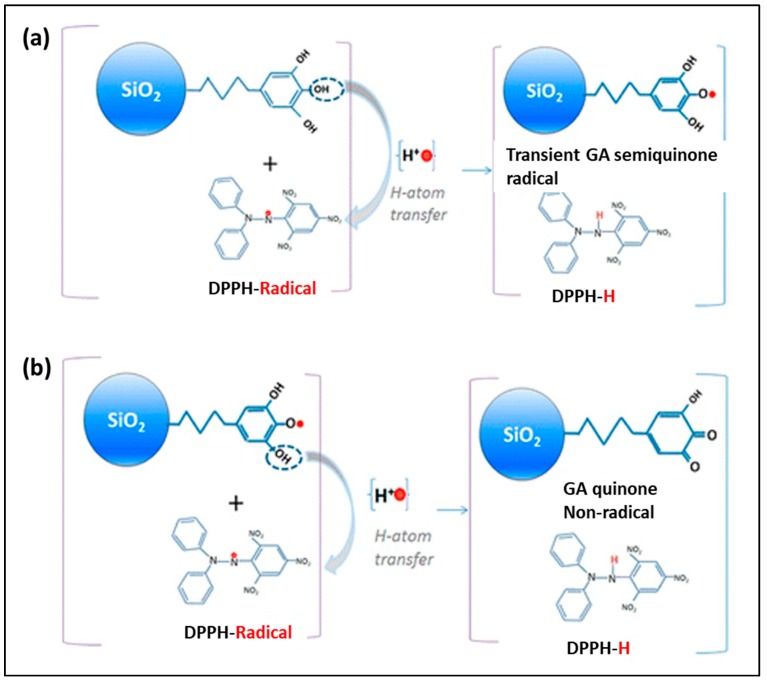
(**a**) Scavenging of one DPPH radical by SiO_2_-GA nanoparticles via HAT (H-atom transfer) reaction from the GA (Gallic acid) molecule forming a transient GA radical. (**b**) Scavenging of a second DPPH radical by SiO_2_-GA nanoparticles via HAT from the GA semiquinone forming a nonradical GA quinone. The figure was adapted from ref. [30], with permission from © 2012 American Chemical Society.

Silica (SiO_2_) nanoparticles are the material of interest, with diverse applications especially in chemistry, medicine, pharmaceutical, and biomedical applications due to being optically transparent, chemically inert, mechanically stable, biocompatible, and having easy and scalable synthesis techniques. Moreover, precise control of SiO_2_ nanoparticles (SiO_2_NPs) in terms of particle size, porosity, crystallinity, and shape, as well as surface chemistry, enables the modulation of different molecules of interest, using a wide variety of coating procedures for diverse application [40,41]. Immobilization of antioxidant on nanosized SiO_2_ particles can yield added-value hybrid nanocomposites. Deligiannakis et al. fabricated nano-antioxidants using commercially available well-characterized SiO_2_NPs of various sizes (8−30 nm), which were functionalized by covalent grafting of natural antioxidants, such as GA, on their surface. These nano-antioxidants were evaluated for scavenging of diphenyl-picryl hydrazine (DPPH) radicals (Figure 1). The SiO_2_-GA nanoparticles perform two types of radical scavenging reactions: (i) rapid H-atom transfer (HAT) and (ii) secondary/slow radical−radical coupling reactions. All SiO_2_NPs perform fast HAT reactions at stoichiometry ratios (n_fast_ = 2), comparable to that of pure GA [30]. Mesoporous SiO_2_NPs (MSN) functionalized with morin (2′,3, 4′,5,7-pentahydroxyflavone, a flavonoid) was also evaluated for its antioxidant potentialities as HO• scavenger and ^1^O_2_ quencher. The nanoantioxidant composite showed one magnitude lower ^1^O_2_ deactivation than morin in homogeneous solvents and lipid membranes, which in contrast exhibited a synergic effect on the antioxidant property against HO• and the effect is proportional to the concentration of morin adsorbed [41]. Similarly, mesoporous poly(tannic acid) (TA) crosslinked SiO_2_NPs composites were also tested for the antioxidant properties by preparing the nanocomposite via varying concentration of TA (50–1000 mg) and reaction times (2–24 h). Particle sizes were between 237–445 nm, solely dependent on the TA content and reaction time, conversely downward thermal stability trend of the composite. Maximum yields of the composite, as well as the largest surface area (872 m^2^/g), were obtained for 12 h and 2 h reaction periods, respectively, for the highest (1000 mg) TA contents, which was also proved as the effective antioxidant materials activity with 14 ± 0.3 mg mL^−1^ total phenol content in term of GA equivalency, and 68 ± 6 mM Trolox (a vitamin E analogue) equivalent g^−1^ [42].

**Figure 2:**
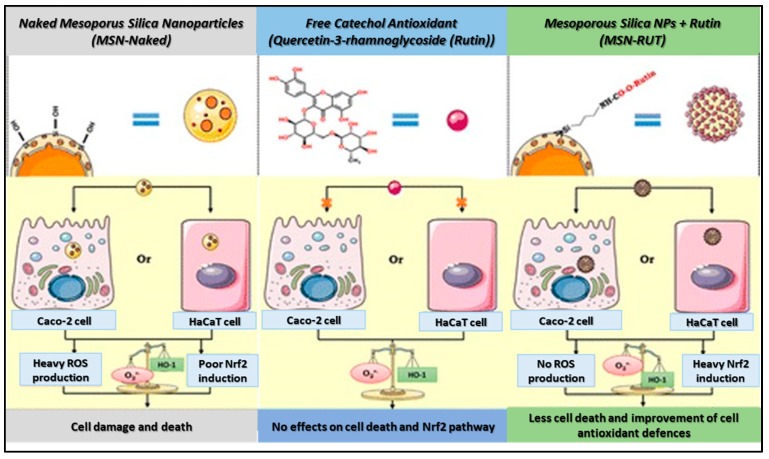
Influence of naked MSNs, free catechol antioxidant (Rutin), and MSNs-RUT on ROS production or Nrf2 induction and consequent cell death. Adapted from Ref. [43] with permission from © 2016 American Chemical Society.

On the other hand, MSN covalently coated with two phenolic antioxidants, namely, caffeic acid (MSN-CAF), or rutin (quercetin-3-*O*-[α-l-rhamnosyl-(1 → 6)-β-d-glucopyranoside]) (MSN-RUT), were investigated for their antioxidative stress features. From a structural point of view, these phenolic antioxidants possess ortho- and meta-hydroxyl groups in the catechol structure, hence being able to scavenge or donate hydrogen atoms to free radical in order to prevent ROS formation and biological damages. Two cellular models, such as the intestinal Caco-2 and the epidermal HaCaT cell lines, were used for the evaluation of ROS production, activation of the Keap1-Nrf2 pathway, and cell death by the free and functionalized MSNs (Figure 2). Antiradical properties of free or grafted antioxidant molecules were also evaluated by using the antiradical test (oxygen radical absorbance capacity (ORAC)). The ORAC test for MSN-CAF or MSN-RUT at 30.3 μg/mL indicates that both particles had a Trolox equivalent level much higher than that of naked nanoparticles or amino-propyl functionalized silica nanoparticles (MSNNH_2_), in addition antiradical function of MSN-RUT was found to be 3.7 times higher than that of MSN-CAF [43].

### 2.2. AuNPs Nanoantioxidant

Gold nanoparticle (AuNP) is the most studied nanoparticle owing to its unique therapeutic activity, inert, and nontoxic nature, therefore has gained huge attention in different field of applications including pharmacological sectors and biomedical fields. [44]. AuNPs obtained from diverse synthesis procedures are also established as effective antioxidant [26]. However, to achieve more advantages in antioxidant/antiradical activities, AuNPs are either immobilized on natural compounds or functionalized with natural antioxidants, as well as other synthetic compounds [45,46]. For example, AuNPs immobilized on cellulose fiber, the unbleached kraft paper, were tested for the DPPH radical scavenging ability in both light and dark conditions. UBK-AuNPs composites showed significantly improved DPPH radical scavenging activity compared to pure fiber composites due to the catalytic function of AuNPs, and interestingly, the composite showed a slightly higher scavenging rate in the presence of light (86.05% ± 0.009) than in darker conditions (77.86% ± 0.006) [47]. In another experiment, polyethylene glycol (PEG) coated AuNPs were functionalized with the antioxidant, salvianic acid A from the composite Au@PEG3SA, which enhanced the DPPH radical-scavenging rate nine times greater than that for the salvianic acid A monomer only [46]. The kinetic behavior of the radical-scavenging activity exerted mainly through scavenging oxygen-centered free-radicals and other ROS was evaluated, both in vitro and in vivo, by means of stopped-flow analysis, laser-scanning confocal microscopic observation, and thio-barbituric acid reactive substances assay, and the obtained results suggested that antioxidant-functionalized AuNPs hold enhanced kinetic effect in ROS scavenging in living cells [46].

**Figure 3:**
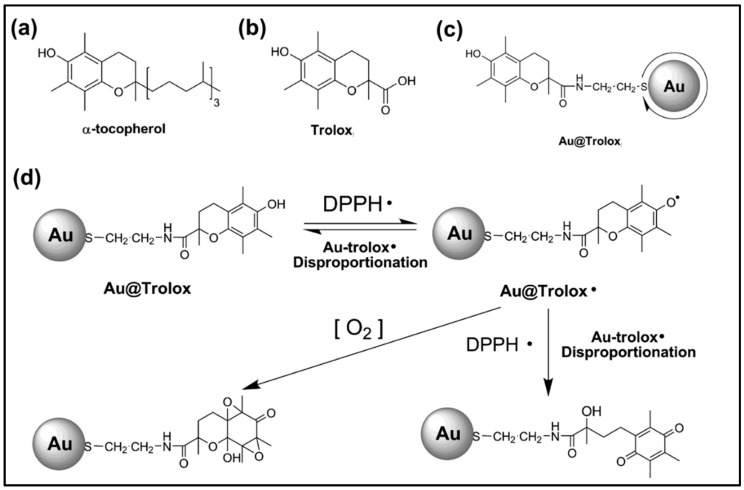
Molecular structure of (**a**) alpha-tocopherol, (**b**) Trolox, (**c**) Trolox functionalized AuNPs, and (**d**) schematic representations of the reaction pathways of Au@Trolox with DPPH• radical. Adopted with permission from Ref. [48]. Copyright Hindawi, 2015.

Trolox functionalized AuNP (Au@Trolox) was synthesized by Au-S bonding, assisted by AuNPs and thiol ligand from Trolox (Figure 3). This Au@Trolox was evaluated for DPPH radical scavenging experiments, which demonstrated enhanced antioxidant activity equal to about eight times higher radical scavenging activity than that of Trolox monomer, and the principle behind this higher activity is supposed to be the organized and ordered assembly of Trolox ligands on the surface of AuNPs (Figure 3) [48]. A combinational study of dietary phytochemicals such as 3,6-dihydroxyflavone, lutein, and selenium methyl selenocysteine, as well as nanotech reinforcement of this phytochemical (AuNPs embedded), were evaluated for DPPH, OH, H_2_O_2_, and NO radical scavenging assays. The triplet combination showed the enhanced radical scavenging activity compared to the native dietary nutrients, as well as single combination at the same concentration level. AuNPs embedded 3,6-dihydroxyflavone showed 72.04% inhibition of DPPH, 70.01% OH, 71.08% H_2_O_2_, 69.01% of NO, which are greater than their native 3,6-dihydroxyflavone (64.21%, 62.11%, 60.11%, and 61.24%, respectively). The inclusion of AuNPs embedded 3,6-dihydroxyflavone with other dietary nutrients, lutein and selenium methyl selenocysteine, further increased the maximum inhibition of 87.13%, 85.11%, 83.10%, and 84.02% for DPPH, OH, H_2_O_2_, and NO, respectively, with overall enhancement in the antioxidant activity of 29.23%, 26.61%, 25.45%, and 26.07% [10].

### 2.3. Silver Nanoparticles (AgNPs) Nanoantioxidant

AgNPs have innumerable applications in medical sciences, due to their profound features. In this context, AgNPs are synthesized via different routes, among which the new green routes of synthesis can provide non-toxic and environmentally benign capping agents [49]. Different green synthesis techniques using different capping agents, including lignin capped silver nanoparticles (LCSN) (grafted on lignin which were highly crystalline with spherical morphology and of size 10–15 nm) [50], *Sida cordifolia* leaf extract induced biogenic synthesis of AgNPs in presence of sunlight [51], aqueous extract of *Clerodendrum phlomidis* L. leaves [51], Seabuckthorn (SBT) leaves extracts employed AgNPs (SBT@AgNPs) [52], and poly(vinyl alcohol) embedded AgNPs (PVA-AgNPs) [53], have been found to have antioxidant activity. LCSN was evaluated for the antioxidant activity, and the half maximal inhibitory concentration (IC50) value for free radical scavenging of DPPH was found to be 3.36 mg/mL, while the standard drug, fluconazole, did not show any antioxidant activity at the same concentration [50]. On the other hand, bio-functionalized AgNPs exhibited greater antioxidant activity in terms of reducing power and free radical scavenging activity. AgNPs showed increased reducing power on phosphomolybdate and ferric ions, as well as higher scavenging activity of superoxide and DPPH radical compared to the extract as well as standard. This increased DPPH and superoxide radical scavenging profile indicates the effective inhibition of free radicals by biosynthesized AgNPs and postulates may be due to different factors, such as capping agents (phytochemicals) on the surface of biosynthesized AgNPs, dispersion, and smaller size (~20 nm). In addition, DPPH radical scavenging activity of AgNPs was found to be dose-dependent and the antioxidant activity is proportional to the dose [51]. SBT@AgNPs also showed dose dependent DPPH radical scavenging abilities, which was increased with the gradual increment of their concentrations (5–25 µg/mL). SBT@AgNPs exhibited more than ten-fold increased DPPH radical scavenging activity compared to the plant extract, and the enhanced activity can be attributed to the presence of phytochemicals of SBT leaves extracts in the nanoparticles as capping agents. Hence, the presence of these phytochemicals and Ag ions result in the antioxidant activities through HAT and single electron transfer mechanisms simultaneously [52].

### 2.4. Iron-Oxide Magnetic Nanoparticles (Fe_2_O_3_NPs) Nanoantioxidant

Antioxidant property of the Fe_2_O_3_NPs has already been explored and the principle is based on the neutralization of free-radical by the transfer of an electron [54]. However, the tailoring of Fe_2_O_3_NPs via different strategies including coating with carbon [55], carboxymethyl-inulin [56], and poly(GA) [57], surface functionalization with natural antioxidant (GA) [27], as well as curcumin in magnetic-silk core-shell nanoparticles [58], was successfully accomplished. These tailored Fe_2_O_3_NP composites showed better dispersibility and stability and were evaluated for the efficient antioxidant properties, antimicrobial activities, and targeted drug delivery to the specific organs, as well as being analyzed for their cytotoxicity and biocompatibility/hemocompatibility [27,55,56,57,58,59]. Surface-functionalized Fe_2_O_3_NPs with GA by in situ and post-synthesis with an average particle size of 5 and 8 nm, respectively, exhibited a 2–4 fold enhanced IC50 values of DPPH antioxidant assay compared to nonfunctionalized Fe_2_O_3_NPs. The free radical scavenging property is most probably due to electron transfer from Fe_2_O_3_-NP@GA to free radicals located at the central nitrogen atom of DPPH, hence enhanced free radical scavenging for Fe_2_O_3-_NP@GA due to the synergistic effect of Fe_2_O_3_-NP and GA [27]. On the other hand, magnetite nanoparticles coated with GA-shell (PGA@MNPs) polymerized in situ at the surface of the particles in a soft and reagent-free process were tested for the antioxidant capacity in Jurkat cells in the presence of H_2_O_2_ as ROS, along with hemocompatibility and blood cell viability experiments. No interaction of PGA@MNPs with whole blood cells were established, rather, a significant decrease was observed of the oxidative stress mediated by H_2_O_2_. The in vitro tests revealed that PGA@MNPs are not only biocompatible, but also bioactive [57].

### 2.5. Cerium Oxide Nanoparticles Nanoantioxidant

Cerium oxide nanoparticles (CNPs) can scavenge the ROS/RNS and perform as antioxidant enzyme-mimetics, which is mostly reliant on the inherent physicochemical properties of the material at nanoscale level, oxygen absorbing and releasing capability, and the relative thermodynamic efficiency of redox cycling between Ce^3+^ and Ce^4+^ ions on the surface of CNPs [60]. CNPs have also been successfully used for the in vitro and in vivo treatment of different cancers, including neuroblastoma, the most recently focused one. However, the anti-cancer features of CNPs depend on the induction and accumulation of ROS with simultaneous decrease in antioxidant enzyme levels [61]. Curcumin possess anti-cancer properties, therefore, combination of CNPs and curcumin in one formulation may result in increased physiological activity. In an experiment, Kalashnikova et al. explored the anticancer properties of curcumin loaded nanoceria (CNP-Cur) and dextran-nanoceria (Dex-CNP-Cur) in MYCN-amplified and non-amplified cell lines of neuroblastoma models. Dex-CNP-Cur were found to evoke considerable cell death in MYCN-amplified IMR-32 cells, that is, a 2-fold and 1.6-fold decrease in cell viability for MYCN-upregulated and normal expressing cell lines, respectively, with no or only minor toxicity in healthy cells (compared to untreated cells). Dextran coating of CNPs, therefore, not only help to reduce the cellular viability in cancer cells, but also assisted in preventing the opsonization and clearance of the nanoformulations from the circulation by the phagocytes. Hence, the formulation induces a long-term oxidative stress by CNP assisted accumulation of ROS and increases the local curcumin concentration, stabilizing HIF-1α, and up-regulating the caspase-dependent apoptosis. CNP-Cur and Dex-CNP-Cur formulations induce the neuroblastomas to increase the ROS production and dramatically decrease the ratio of Bcl-2/Bax (Bcl-2 stands for anti-apoptic factors and Bax is apoptosis-inducing gene) which consequently triggers the release of cytochrome C, guide to caspase 3/7 activation, and apoptosis [61].

### 2.6. Dual Nanoparticles Nanoantioxidant

**Figure 4:**
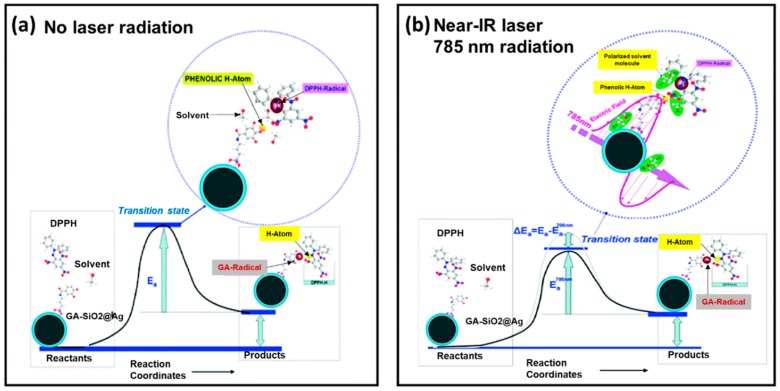
HAT mechanism from the phenolic OH of the conjugated GA of GA-SiO_2_@Ag nanoantioxidant to a DPPH radical under (**a**) no laser irradiation and (**b**) under 785 nm laser irradiation. The HAT mechanism/step proceeds via an activated transient state involving the association between DPPH• and GA. Upon 785 nm laser excitation at near-IR spectral region, a hot-spot was created by the agglomeration of the nanoparticles and a strong vibrational local electric field was produced, which lowers the activation energy (E_a_) by at least 2 kcal mol^−1^. Adopted with permission from Ref. [63]. Copyright The Royal Society of Chemistry, 2016.

Like single nanoparticles, dual nanoparticles are also grafted with antioxidant or formed by the reaction with antioxidants with the intention of achieving higher antioxidant properties. Bimetallic nanocomposites in different combinations, as well as those functionalized with different antioxidant molecules, were evaluated for their ROS or RNS scavenging capacity. Quercetin and GA mediated synthesis of Ag and selenium (Se) based bimetallic nanoparticles (Ag-Se) were accomplished and the stabilized, mono-dispersed Ag-Se bimetallic were tested in vitro for their antioxidant efficiency using azino-bis-ethyl benzthiazoline-sulfonic acid (ABTS), 3-(4,5-dimethylthiazol-2-yl)-2,5-diphenyltetrazolium bromide (MTT) and DPPH assay [62]. The antioxidant activity of the nanoantioxidant was found in the range of 59–62% and 30–66 µg/mL in terms of percentage and IC50 value, respectively. Here, the potentiality of the nanoantioxidant is linked to the redox potential of the capping agent, quercetin, and GA on Ag-Se surface, which allow them to act as strong reducing agents and H^+^ donors and ^1^O_2_ scavengers, and consequently justify the nanocomposite as a potent antioxidant [62]. SiO_2_-coated AgNPs functionalized with GA (GA-SiO_2_@Ag) shows plasmon enhanced proton-coupled electron transfer (PCET) at near-IR wavelengths (700–1100 nm). The increased PCET to the near-IR spectral region at 785 nm laser incitement is due to the nanoparticle’s agglomeration by the grafted GA molecules, and consequently, the formation of hot-spots of SiO_2_-coated AgNPs. Upon laser excitation, hot-spots provoke a notable increase of HAT from GA to DPPH radicals due to decreasing of the bond dissociation enthalpy (BDE) of the phenolic OH moieties, thus significantly accelerating the DPPH• scavenging kinetics (Figure 4) [63].

**Figure 5:**
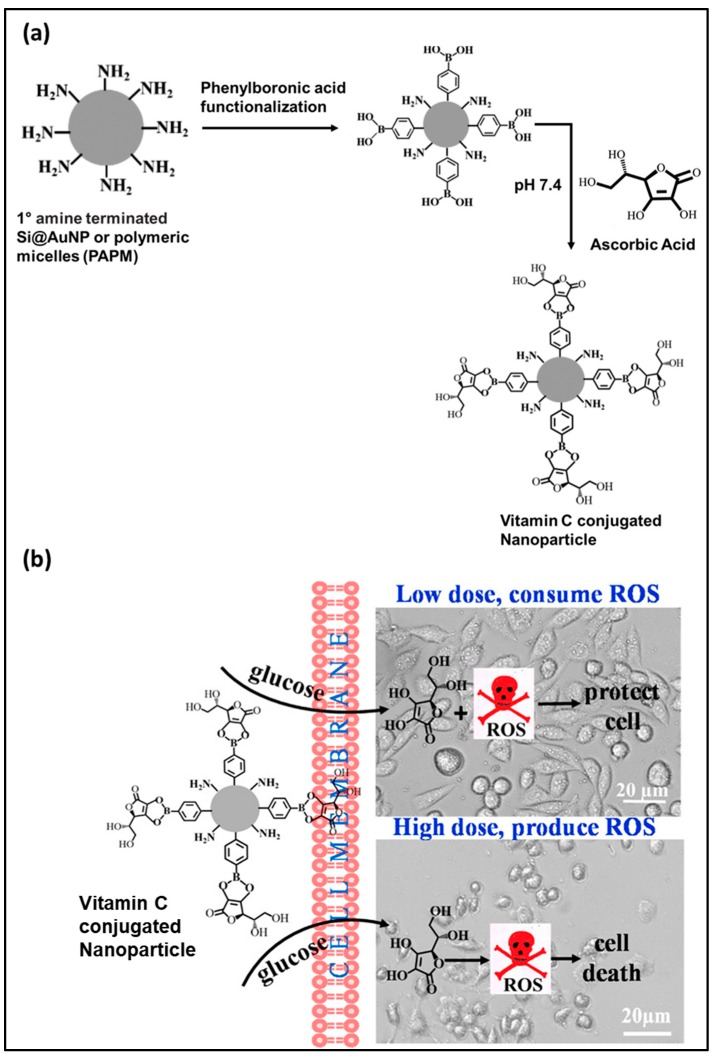
(**a**) Schematic presentation on the synthesis of vitamin C conjugated nanoparticles (Si@AuNP or PAPM) and (**b**) cellular oxidative stress at micro and millimolar concentration of vitamin C. Adapted with permission from Ref. [32]. Copyright American Chemical Society, 2017.

In another experiment, vitamin C conjugated with two different nanoparticles, silica-coated AuNPs (Si@AuNP) and polyaspartic acid-based polymer micelles (PAPM), were studied for glucose responsiveness and efficient targeted delivery within the cell. The fabrication of the nanocomposites involved two steps—firstly, functionalization of the primary amine-terminated nanoparticle (either Si@AuNP or PAPM) with phenylboronic acid, followed by the conjugation of vitamin C to nanoparticle via chelation (Figure 5a). The hydrodynamic size of the resultant Si@AuNP and PAPM are 40–50 nm and 40–80 nm with ∼4–8 wt % and ∼10–13 wt % of vitamin C, respectively. The cellular oxidative stress was evaluated at two different vitamin C concentrations and found that at micromolar concentration it protects the cell, while at millimolar concentration it induces the cell death (Figure 5b) by generating oxidative stress, particularly by producing H_2_O_2_, which disrupts the cellular redox balance [32].

### 2.7. Polymeric Nanoantioxidant

Polymeric nanoparticles, which are mainly composed of synthetic biodegradable polymers, are one of the most promising nanocarriers being developed. Poly(ε-caprolactone) (PCL) is one of the prominent synthetic polymers approved by the US-FDA, which has been utilized for the integration of aqueous extract of *Syzygium cumini* (ASc) seeds via an emulsification/evaporation solvent method. The ASc and PCL-ASc were evaluated by the DPPH radical scavenging capability and ferric reducing antioxidant power assay (FRAP). The study revealed that both ASc and PCL-ASc, even at the very low concentration (100 µg/mL), have almost the same and high scavenging DPPH radicals activity and reducing power in the FRAP assay, meaning that immobilization of ASc in PCL nanoparticle had no influence over antioxidant scavenging activity [64]. Bacterial cellulose (BC), a polymer composed of nanofibers and of biological origin, has also received huge interest due to its excellent film-forming ability, higher water holding capacity, porosity, and most importantly, biocompatibility. BC as a nanocarrier can adsorb spherical nanoparticles comprising of flavonoid silymarin and zein (SMN-Zein). Assembly of SMN-Zein with BC films can form SMN-Zein/BC nanoparticles/nanofibers composites with enhanced wettability and swelling property of BC films, as well as boosted solubility of sparingly soluble silymarin and release from the nanocomposite films. SMN-Zein/BC showed greater DPPH, ABTS%, and superoxide anion scavenging activity compared to free SMN. BC did not exhibit any antioxidant activity; however, its presence prolonged the antioxidant potential of the composites by the slow release of the SMN [65]. The important nanoparticle carriers, corresponding functionalized antioxidants and their remarkable features, are summarized in Table 1.

## 3. Nanogel Entrapped Antioxidant

Regardless of antioxidant integration onto the nanoparticle surface, or antioxidant encapsulation by the nanoparticles itself, nanogel could be an alternative plausible media for the incorporation of antioxidant molecule into the backbone of a polymer towards the effective delivery to increase the systemic bioavailability and controlled release of the antioxidant. In this regard, Wattamwar, et al. have synthesized hydrolytically degradable crosslinked polymers, such as poly(β-amino esters) (PβAEs) hydrogel for the simultaneous controlled release of two antioxidants with different antioxidant functions and therapeutic potentials. Here, acrylate functionalized quercetin multiacrylate and curcumin multiacrylate were synthesized and incorporated into the polymer backbone from poly(antioxidant β-amino ester) (PAβAE) hydrogels. Quercetin and curcumin PAβAE hydrogels took 5–6 h for the complete degradation, while the control sample (hydrogel with no antioxidant) was degraded quickly within 150 min. Upon degradation, the active antioxidants were released into the human umbilical vein endothelial cells and suppressed H_2_O_2_-induced oxidative stress, resulting in improved material biocompatibility and maintaining cellular viability [70]. However, the bulk crosslinking approach in the typical nanoparticle synthesis methods (e.g., o/w emulsion polymerization), where water is being used as a dispersing media, poses some limitations due to the fast reaction kinetics and degradation properties of PβAE. To mitigate the limitations, Gupta et al. formulated the quercetin conjugated PβAE nanogel by a single-phase reaction–precipitation method in an organic solvent. Here, self-stabilized suspension of PβAE nanogel was formed via covalent linking between acrylated quercetin and a secondary diamine. However, to increase the aqueous stability and decrease the first pass metabolism rate, a post synthesis modification of spherical nanogels was achieved by covalent PEGylated coating. The active quercetin antioxidant was released uniformly with negligible burst release over 48 h upon the degradation of the nanogels under physiological conditions retaining antioxidant activity [2].

## 4. Hollow Nanosphere Tagged Nanoantioxidant

**Figure 6:**
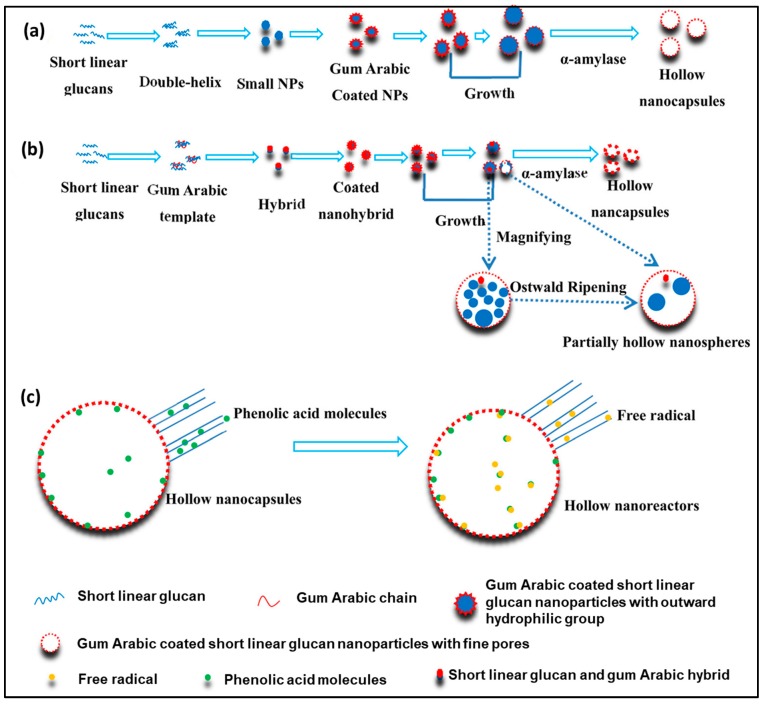
Graphical presentation of the fabrication of hollow gum arabic coated short linear glucan nanocapsules (**a**) and in situ short linear glucan/gum arabic hybrid (**b**); loading of phenolic acid followed by free radicals scavenging activity into the hollow nanoreactors (**c**). Adopted with permission from Ref. [71]. Copyright American Chemical Society, 2017.

Hollow nanospheres have some typical attributes, such as large surface-to-volume ratio, low density, short solid-state diffusion length, and good surface permeability, which have attracted great attention to be considered as drug delivery vehicles. Li et al. [71] developed a green amylolysis technique for the fabrication of hollow gum arabic coated short linear glucan@ nanospheres and hollow in situ short linear glucan/gum arabic hybrid nanospheres by using starch nanoparticles and natural gum arabic as the core-shell materials, respectively (Figure 6). These gum arabic coated short linear glucan core-shell nanospheres were utilized for the loading of phenolic acid and allowed easy access to free radicals to the interior via its porous nanoshell formed by amylase activity and reacting with phenolic acid. Thus, the hollow nanocomposite improves the free radicals scavenging activity and simultaneously hides the inherent undesired odors, taste of phenolic acid, and improves stability at extreme conditions, such as UV light and heating [71].

**Figure 7:**
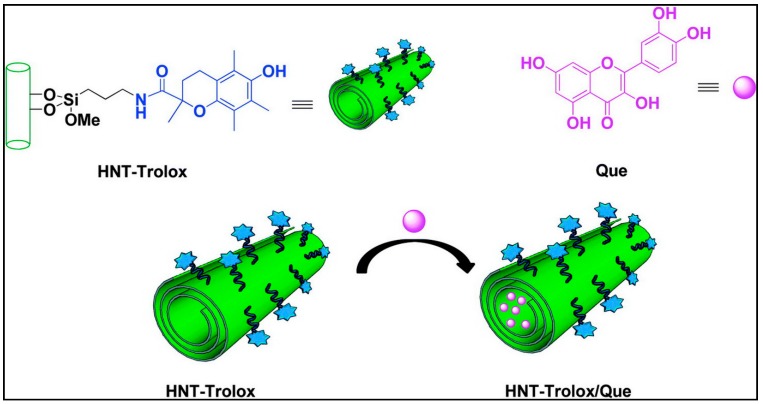
Graphical illustration of quercetin loading into HNT–Trolox. Adapted with permission from Ref. [72]. Copyright The Royal Society of Chemistry, 2016.

A nanoantioxidant based on halloysite nanotubes (HNTs) with selective grafting of Trolox on the external surface and inner loading of quercetin into the lumen of the tube was fabricated to form HNT–Trolox/Que bi-functional nanoantioxidant (Figure 7). These HNT–Trolox/Que were checked for scavenging activity against transient peroxyl and persistent DPPH radicals compared to the corresponding mono-functional HNT–Trolox and HNT/Que analogues. Both HNT–Trolox and HNT/Que showed effective capability to quench both peroxyl and DPPH radicals while protecting organic materials from autooxidation. However, the synergistic activity of the HNT–Trolox/Que nanoantioxidant enhanced the scavenging of peroxyl and DPPH radicals at 35% higher in acetonitrile and 65% in chlorobenzene, as compared to the expected cumulative contributions of NHT-Trolox and NHT/Que, which is in fact based on the prompt reaction of the externally exposed Trolox with free radicals followed by the slow and prolonged release of quercetin from the HNT lumen for the regeneration of Trolox [72].

## 5. Nanoparticles Mediated Antioxidant Encapsulation and Delivery

Most active molecules are chemically unstable and prone to enzymatic/chemical degradation, which can result in incomplete absorption and excretion from body. This can lead to insufficient concentration of bioactives (low bioavailability) to produce a meaningful therapeutic outcome. Consequently, the need for enhancing the therapeutic value of various water soluble/insoluble bioactive molecules by improving bioavailability, solubility, as well as controlled and target specific delivery, is urgent [73,74]. Many promising delivery systems have already been developed to enhance the therapeutic efficiency of bioactives. In this aspect, particulate carriers, such as NPs, have already revealed great potential for protecting active molecules from premature degradation, while achieving temporally controlled delivery of bioactives at specific body sites. Entrapment of biologically active agents within NPs can be achieved using polymers with well-defined physical and chemical properties in order to protect sensitive bioactive components against external factors before reaching the target and releasing in a controlled and predictable manner. Polymers used for nanoencapsulation may be classified either as synthetic or natural polymers.

Synthetic polymeric NPs are promising drug carriers that can be beneficial by encapsulating and gradually releasing the compounds. Consequently, the absorption of active compounds by active endocytosis in the small intestine is enhanced, retaining the biological activity, improving the targeting, and increasing the bio-accessibility [75]. Among synthetic polymeric NPs, poly-D,L-lactide (PLA) and poly(lactic-co-glycolic acid) (PLGA) are the most commonly used biodegradable polymers, approved by the U.S. food and drug administration (FDA) and the European medicine agency (EMA) as safe for parenteral administration. In fact, they present low toxicity, are physically strong, highly biocompatible, and have been extensively studied as delivery vehicles for drugs, proteins, and various other macromolecules such as DNA, RNA, and peptides. Although PLA and PLGA are the most popular among the various available biodegradable polymers because of their long clinical history, favorable degradation characteristics, and possibilities for sustained drug delivery at desirable doses, other synthetic polymers, such as PCL and PβAEs, have also been used as nanocarriers of antioxidants. Moreover, natural polymers, such as proteins, polysaccharides, chitosans, gum arabic, and polypeptide polymers are also used for the encapsulation of various antioxidant molecules effectively and exert some competitive advantages in terms of biocompatibility, bioavailability, and biosafety over synthetic polymers (Table 2). However, the synthesis procedure of natural polymeric nanoparticles involves complex steps compared to synthetic one, and may offer an alternative for the preparation of nanocarriers [76]. Furthermore, natural polymers are less commonly used due to their inconsistent purity, and b) the requirement of crosslinking step, which can adversely affect the encapsulated materials [77] and fast release of encapsulated therapeutic agents compared to synthetic polymers. However, they are preferred to synthetic polymers in having milder formulation conditions, such as the nature of solvents required for dissolving them [78]. Moreover, liposomes, spherical vesicles of artificially-prepared phospholipid bilayers, are also being used as antioxidant delivery vehicles (Table 2). Both lipophilic and hydrophilic antioxidants can be loaded into it due to its amphiphilic structure (liposomal bilayer and aqueous core) and are mostly favored for the encapsulation of water insoluble antioxidants, as well as water soluble antioxidants and antioxidants enzymes [76,79]. In this section, we have discussed the nanoparticle aided antioxidant delivery in respect to different nanoparticles/combination of nanoparticles, as well as nanoencapsulation approaches.

### 5.1. Polymeric Encapsulation and Delivery

#### 5.1.1. Poly-d,l-lactide-Based Nanoparticles

Roussaki et al. [80] investigated the nanoencapsulation of a naturally-occurring antioxidant, aureusidin, in PLA NPs. The NPs were prepared by an emulsification-solvent evaporation technique in the presence of various amounts of aureusidin (20% and 60%) and particles of between 231–376 nm in size were obtained. The size of NPs decreased during 60 days of storage at 4 °C in an acidic aqueous solution due to hydrolysis of the polymeric matrix and diffusion of the encapsulated compound towards the aqueous environment. The encapsulation efficiency was in all cases over 68%, reaching even 98%, for drug loading equal to 60%. An emulsification-solvent evaporation technique was also used [81] to develop PLA NPs containing ursolic acid and evaluate the radical scavenging activity over hypochlorous acid (HOCl) and cytotoxicity on erythrocytes and tumor cells. Ursolic acid is a natural antioxidant with anti-inflammatory, antihyperlipidemic, antidiabetic, hepatoprotective, neuroprotective, antiobesity, and anticancer properties. The results showed that the nanoencapsulation process led to drug amorphization and prolonged ursolic acid release. The release profile was biphasic with a high initial release, followed by a sustained release: in the first 8 h, about 30% of ursolic acid was released, and after 120 h, about 60% of the antioxidant was released from PLA NPs. Furthermore, NPs in the suspension demonstrated to be physically and chemically stable at room temperature and at pHs 1.3 and 6.5 for 180 days. Additionally, NPs exhibited low hemolytic potential and maintained the antioxidant potential and antitumor activity. The antioxidant activity of free and nanostructured ursolic acid was evaluated based on its ability of HOCl inhibition, and after 72 h, the ursolic acid-loaded NPs demonstrated similar activity to plain antioxidant in all tested concentrations. On the other hand, antitumor activity was evaluated on melanoma cells (B16–F10). The results showed that after 72 h, the cytotoxicity of ursolic acid-loaded NPs was improved, and in all tested concentrations the cell viability was below 50%. PLA NPs can be also used to encapsulate quercetin for improving aqueous solubility and stability. PLA encapsulated quercetin showed higher aqueous solubility, sustained release, and identical antioxidant activities as of free quercetin. The drug release involved two phases; an initial burst release where 40–45% quercetin was released within 0–0.5 h, attributed to surface absorption, followed by a slow and sustained release where 87.6% of the antioxidant was released after 96 h, attributed to the diffusion of the quercetin entrapped within the core of the NPs [82].

#### 5.1.2. PLGA-Based Nanoparticles

Quercetin-loaded PLGA NPs were evaluated for the increased bioavailability and therapeutic effects. In vitro drug release studies showed that PLGA NPs were capable of maintaining a sustained drug release profile of quercetin in comparison with pure quercetin. Approximately 56% and 65% of quercetin was found to be released in phosphate buffer from the best two formulations. Moreover, the NPs also showed better antioxidant and diuretic activities in comparison to pure quercetin, hence suggesting that PLGA NPs can be a potential candidate for quercetin delivery [83]. In another study, Betbeder et al. prepared curcumin-loaded PLGA-based NP and checked the antioxidant activity both in cellular and acellular ways. Curcumin is a hydrophobic polyphenolic compound that possesses a wide range of beneficial biological and pharmacological properties, including anti-inflammatory, antioxidant, antimicrobial, and anticarcinogenic, which are, in contrast, hindered by its poor water solubility, short biological half-life, and the consequent lower bioavailability in both plasma and tissue. Hence, to increase the bioaccessibility and antioxidant activity of curcumin, these researchers found that curcumin-loaded NP shows 20–50 times greater antioxidant activity in epithelial cells compared to free curcumin. This is due to the efficient entry of curcumin into airway epithelial cells, mediated by NP endocytosis and the curcumin-NP’s bypassing of the cells’ efflux pump. Both antioxidant and direct antinitrosant effects of curcumin-NP were found to be greater when curcumin was formulated in PLGA-NP, probably owing to the formation of a nano-environment that concentrates and facilitates interactions of the curcumin with ROS and RNS [84]. In another experiment, GA was encapsulated in PLGA nanoparticles (NP-PLGA-GA) or polysorbate 80 (PS80)-coated PLGA nanoparticles (NP-PLGA/PS80-GA) by emulsion solvent evaporation method. Coated NPs (NP-PLGA/PS80-GA) showed a slower release of the polyphenol antioxidant than non-coated PLGA NPs, suggesting that PS80 coated NPs have an additional barrier to GA release, hence they could be advantageous to reach the brain. Antioxidant profile was determined by in vitro colorimetric measure of radical cation ABTS•^+^. In higher GA concentrations, 24 h NP-PLGA-GA showed a similar percentage of radical inhibition to free GA, which might be due to the burst effect of PLGA NPs, while the decreased antioxidant activity after 48 h refers the slow drug release. In the same conditions, NP-PLGA/PS80-GA produced the least antioxidant effect. Moreover, cytotoxicity evaluation of the compounds on erythrocytes was assessed. NP-PLGA-GA in all analyzed concentrations demonstrated lack of hemolysis, while NP-PLGA/PS80-GA were cytotoxic only in higher concentrations [54].

#### 5.1.3. Poly(ε-caprolactone)

PCL is a semi-crystalline polyester that is biodegradable and biocompatible. These properties have led to the approval of several PCL drug-delivery devices and implants by the FDA. It presents high permeability to many bioactives, lack of toxicity, and a slow rate of degradation in-vivo compared with other biodegradable polyesters [85]. Due to these properties PCL can be exploited in the manufacturing of antioxidant-controlled release formulations. For instance, PCL has been used for encapsulating a mixture of quercetin and biapigenin by solvent displacement method. These two antioxidant and neuroprotective compounds were isolated from *H. perforatum* and the encapsulation of these bioactives resulted in NPs ranging from 185 to 250 nm in size. Along with the physical and morphological parameters of the NPs, the in vitro release profile, the antioxidant activity of the compounds, and the potential protective effect of quercetin-biapigenin against *t*-BOOH-induced oxidative stress in HepG2 cells was also studied [86]. High encapsulation efficiency, small size (~200 nm), a sustained release (75% after 8 h), and the reproducibility of the production method made quercetin-biapigenin loaded PCL NPs a suitable candidate for the treatment of neurodegenerative diseases, such as Alzheimer’s and Parkinson’s disease. In addition to the uninfluenced antioxidant activity after encapsulation of the compounds determined by the DPPH-radical scavenging (5.73 ± 1.20 EC50 µg/mL) and Iron (II) chelating activities (23.50 ± 0.55 EC50 µg/mL), the potential protective effect of quercetin-biapigenin against *t*-BOOH-induced oxidative stress in HepG2 PCL-loaded NPs was also shown.

#### 5.1.4. Poly(β-amino esters)

PβAEs are obtained from the co-polymerization of diacrylate and amine molecules. They have been the subject of some studies employing them as delivery systems due to their biocompatibility, biodegradability, and in vitro as well as in vivo cytocompatibility properties [87]. As an example, Gupta et al. developed an alternative way of quercetin integration in the polymeric prodrug carriers by incorporating the polyphenol into the backbone of the polymer matrix, PβAEs nanogels, via reaction of 4–5 acrylate groups of acrylated quercetin with the secondary diamine. Quercetin conjugated PβAE nanogel carriers would allow multiple delivery routes (oral, intravenous, inhalation) as well as providing a uniform and slow release of the intact quercetin molecule over extended periods (up to 48 h) [2].

#### 5.1.5. Polyanhydride Nanoparticles

**Figure 8:**
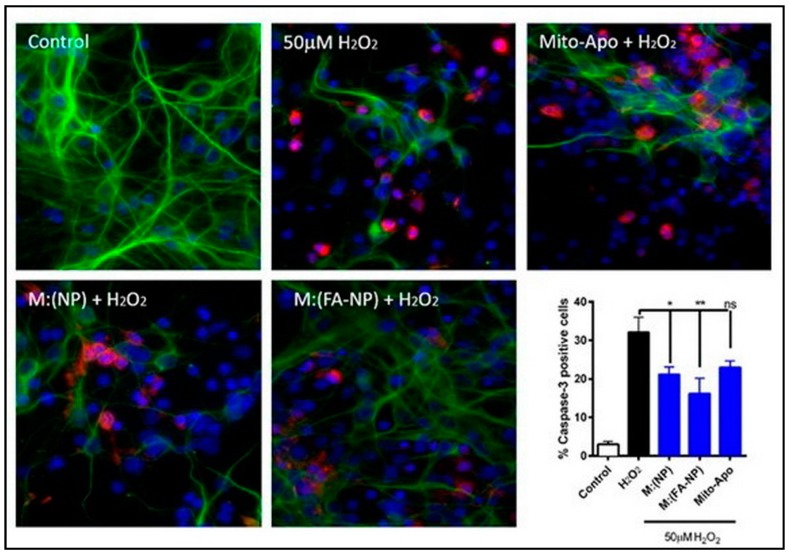
Efficacy of Mito-Apo-encapsulated nano-formulations on primary cortical neurons. Neurons were pre-treated with the various nano-formulations for 24 h and challenged with H_2_O_2_ for 1.5 h. Neurons were stained for β-III tubulin (green) and cleaved caspase-3 (red). Cell death was quantified by the presence of cleaved caspase-3 and by the reduction in neurite length. Caspase-3-positive cells were quantified as shown in the graph. * = *p* < 0.05; ** = *p* < 0.01. Adapted with permission from Ref. [88]. Copyright Elsevier, 2003.

In another study, the intracellular delivery of the mitochondria targeted antioxidants, such as apocynin (Mito-Apo) encapsulated in the biodegradable polyanhydride nanoparticles functionalized with folic acid (FA) or not, were evaluated for oxidative stress-induced mitochondrial dysfunction and neuronal damage in a dopaminergic neuronal cell line, mouse primary cortical neurons, and a human mesencephalic cell line. Polyanhydride nanoparticles provided a slow and controlled erosion of the particles, enabling a sustained release of the encapsulated cargo, whereas the functionalization with FA improved nanoparticle internalization, with 32% of N27 cells internalizing FA-NP compared to only 25% of cells internalizing NP. Mito-Apo-encapsulated nano-formulations (with or without FA) and Mito-Apo alone limited the H_2_O_2_-induced increase in cleaved caspase-3 immunostaining to 1.5-, 2.0-, and 2.5-fold, respectively. Together, these results indicate that M:(FA-NP) effectively inhibited H_2_O_2_-induced cell death, especially compared to M:(NP) or Mito-Apo alone (Figure 8) [88].

#### 5.1.6. Prodrug Approaches

As therapeutic agents for various ROS-associated inflammatory diseases, Kwon et al. [89] developed poly(vanillin oxalate) (PVO) as a polymeric prodrug of vanillin, which covalently incorporated vanillin in its backbone and released it during its hydrolytic degradation. Vanillin is a major component of natural flavoring agent vanilla, is an aromatic aldehyde containing hydroxyl group, and is known to have potent antioxidant and anti-inflammatory activities. Vanillin shows minimal or insufficient therapeutic effects because the orally administrated form rapidly decomposes in the upper digestive tract and the intravenously injected vanillin swiftly clears from blood circulation. The dual stimuli-responsive polymeric prodrugs of PVO showed pH-dependent hydrolytic degradation kinetics and vanillin release behavior. They found a half-life of hydrolysis of ~24 h at pH 7.4 and ~15 h at pH 5.5 of PVO NPs and they showed that the majority (~80%) of vanillin was released within 36 h at pH 5.4, while significantly less (40%) vanillin release occurred at pH 7.4 due to the acid-cleavable acetal linkages. Furthermore, PVO NPs showed highly potent antioxidant and anti-inflammatory activities, along with an excellent biocompatibility with PVO NPs at doses less than 500 µg/mL having tremendous potential as therapeutics for oxidative stress-associated inflammatory diseases.

### 5.2. Polysaccharide-Based Nanoparticles

#### 5.2.1. Chitosan Originated Nanocarrier

Chitosan has been widely used to produce nanoparticles as sole material or in combination with others [77]. One of the advantages of using chitosan is its mucoadhesive property, which helps to enhance the targeted delivery of nanoparticles in mucosal surfaces such as nasal and intestinal epithelium [90]. Nayak et al. prepared chitosan nanoformulations, encapsulating antioxidants such as ascorbic acid (Vitamin C), α-tochopherol (Vitamin E), and catechol, along with green synthesized AgNPs by ionotropic gelation method to overcome the challenges associated with encapsulation process, such as initial burst release, instability, and incomplete release, as well as targeted drug delivery to breast cancer affected cells to scavange the produced large amount of ROS. The nanoformulations showed high encapsulation efficiency up to 76% with higher antioxidant activity than their base materials and were found to be highly hemocompatible. The nanoformulations also added to the benefits by providing prolonged and sustained drug release only at the targeted site, with minimal side effects to the normal cells [91]. Curcumin encapsulated by nano and pickering emulsion stabilized by chitosan-tripolyphosphate (CS-TPP) nanoparticles were evaluated for their radical scavenging activity and found higher results compared to free curcumin, which dictates the protective effect of the emulsion systems on antioxidant activity of curcumin bioactive compounds [92]. Quite interestingly, Pu et al. demonstrated that the encapsulation of curcumin antioxidant via smart nanoparticles and the release triggered by the both oxidative stress and reduced pH to the inflamed tissues to inhibit the overproduction of ROS/RNS produced by the lipopolysaccharide (LPS)-stimulated macrophage. In the experiments, authors have used pH-responsive NPs made of N-palmitoyl chitosan (NPCS) that bears a hydrophobic Cy3 moiety, while make the carrier simultaneously responsive to pH and oxidative stress, also encapsulated by poly-(1,4-phenyleneactone dimethylene thioketal) (PPADT). PPADT contains thioketal linkages, which in response to ROS can be selectively degraded into hydrophilic fragments while remaining stable in an environment containing an acid, base, or protease [93].

In another study, Kaur et al. [94] formulated catechin hydrate (CH)-loaded nanoparticles by ionic gelation method by drop wise infusing the anionic polymer (tripolyphosphate) into drug loaded polymeric cationic solution of chitosan, finally leading to the three-dimensional lattice formation. Catechin loaded nanoparticles (CHNPs) showed higher DPPH (67.01 ± 0.15%) scavenging activity as compared to pure CH (65.69 ± 0.34%), similarly better anti-radical and scavenging activity of nitric oxide (NO) (46.12 ± 0.11%) and H_2_O_2_ (36.31 ± 0.31%)) as compared to pure CH (NO scavenging = 42.31 ± 0.14% and H_2_O_2_ scavenging = 38.12 ± 0.11%). This improved antioxidant activity dictates the greater efficiency and therapeutic index of encapsulated CH. Moreover, the encapsulation process ensures the slow release of CHNPs (83%, 12 h) with sustainability until 12 h and less cytotoxicity than CH [94]. Chlorogenic acid (CGA), another polyphenolic antioxidant, was encapsulated into chitosan nanoparticles by ionic gelation method, and ensures that the free radical scavenging activity of CGA was restrained and the antioxidant activity of CGA (IC50 value 92 ± 5 µg/mL) is better than unencapsulated CGA (IC50: 89 ± 3 µg/mL) [95]. Chitosan/DNA co-assemblies, due to their opposite charge, were interestingly used to fabricate nanocarriers for the efficient encapsulation of astaxanthin, a carotenoid with strong antioxidant properties. The efficient encapsulation of astaxanthin in the nanocarriers is due to the directly react with stacked bases of DNA via intercalation, groove binding, or electrostatic interaction, as well as entrapment into the hydrophobic micro-domains constructed by chitosan. The obtained transparent and homogenous astaxanthin-loaded DNA/chitosan colloids showed more powerful antioxidant activity by enhancing the cellular uptake in a short time by the Caco-2 cells, improving the cell viability from 49.9% to 61.9%, active against H_2_O_2_-induced oxidative cell damage (cytoprotective effect), as well as greater ROS scavenging efficiency than that of free astaxanthin at the equivalent/same concentration (3.35 nM) [96].

#### 5.2.2. Starch Nanoparticles

CH and some other well-known polyphenols, secondary metabolites in plants, have potential therapeutic properties, but are limited by their low oral bioavailability, poor stability, and intestinal absorption, forced to think about the development of targeted nanoparticle-based carrier system to overcome the physicochemical limitations and increase its stability, bioaccessibility, and control release of the active phenolic phytochemicals [74,94]. In this aspect, four polyphenols: (+)-catechin, (−)-epicatechin, (−)-epigallocatechin-3-gallate, and proanthocyanidins, were loaded with biocompatible starch nanoparticles (SNPs) and antioxidant potentiality was investigated under different conditions of ionic strength, high temperature, and ultraviolet radiation. Polyphenol-loaded SNPs (PSNP) exhibited monodisperse spherical shapes with no obvious aggregation, and retained antioxidant activities under UV radiation, as free polyphenols are sensitive to UV, hence decreasing the DPPH radical scavenging activities, as well as showing greater stability in NaCl, ultraviolet radiation, and temperature treatments in compare to free polyphenols. Moreover, PSNPs are non-cytotoxic at low concentrations to MEF cells, and exhibited a sustained release under different pH conditions, hence proving an effective nanocarrier to protect bioactive compounds against sensitive environments and to control their release [74].

#### 5.2.3. Alginate

Alginate is a natural polymer that can be derived from some species of algae or bacteria such as phaeophyceae, *Azotobacter vinelandii*, and *Pseudomonas* [97]. Biocompatibility, availability, low cost, and simple formulation have made alginate an ideal natural and one of the most commonly used polymers in formulation studies [98] (Krasaekoopt, Bhandari and Deeth, 2003). Alginate nanohydrogels have been used for antioxidant delivery, mostly by using multi-functional cross linker and in combination with other polymers to improve the mechanical properties and bioactive release profiles [99,100]. Das et al. reported development of alginate-chitosan nanoparticles for delivery of curcumin. The size of the produced nanoparticles were about 100 nm and the encapsulation efficiency was improved by addition of pluronic F127 [101,102]. Another study revealed the antioxidant effects of chitosan/alginate nanoparticles loaded with quercetin, the safety, and the improved protective activity of the encapsulated quercetin compared to non-encapsulated quercetin [103].

### 5.3. Protein-Based Nanoparticles

Protein-based nanoparticles have been used for bioactive delivery applications. Proteins are suitable materials for nanoparticles preparation due to their bio-degradability, compatibility, and availability. Furthermore, since proteins are known amphiphilic polymers, they can interact with both hydrophilic and hydrophobic moieties, which widens the range of biomaterials and solvent that can be used in nanoparticle formation. Nanoparticles can be made of various protein sources, including water- soluble (e.g., bovin serum albumin (BSA) or human serum albumin) and insoluble (e.g., zein and gliadin) that have been used extensively to develop controlled release system. The main protein-based materials that have been used for antioxidant delivery are summarized below.

#### 5.3.1. Albumin

Albumin can be used as a protein carrier due to its biocompatibility, biodegradability, and non- immunogenicity. Various binding sites within the albumin matrix provided a high capacity for encapsulating different materials [104]. Furthermore, the presence of charged amino acid units within the primary structure of albumin can induce the electrostatic attraction, facilitating encapsulation of a wide range of molecules [105,106]. Several commercially available sources of albumin, including ovalbumin, BSA, and HAS, have been used for controlled delivery purposes [107,108,109,110]. Glutaraldehyde [111,112], methyl PEG modified oxidized dextran (Dextranox-MPEG), 2,3-butadiene, or formaldehyde have been used as cross-linking agents for albumin [113]. However, these cross-linkers may have undesirable effects on entrapped materials within nanoparticles. Therefore, application of heat is used as a chemical-free approach to solidify albumin nanodroplets [114]. Other approaches, such as surface modification (octyl-modified BSA) [115], changing albumin hydrophobicity, or using lipophilic drugs can also be used as a chemical free approach to induce self-assembly of albumin into nanoparticles [116]. Physical adsorption of other polymers to negatively charged albumin by taking the advantage of ionic interactions between the two oppositely charged polymers can also be applied [117]. Albumin nanoparticles have been commonly used to deliver anti-cancer drugs due to their accumulation in malignant tissues and long half-life [108], or can act as an efficient nanocarrier to deliver hydrophobic antioxidants [118].

#### 5.3.2. Gelatin

Gelatin is a protein derived from collagen and is commercially available as type A or type B [119]. Gelatins are biodegradable, biocompatible, and non-antigenic, and can readily go under surface alteration due to the defined amino acid sequence in their primary structure. Various methods have been used to prepare gelatin-based nanoparticles, including two-step desolvation, simple coacervation, microemulsion, nanoprecipitation, and self-assembly [120]. Gelatin nanoparticles can be obtained with and without crosslinking. However, some problems such as aggregation and less stability were reported for nanoparticles formed without crosslinking [113]. Gelatin nanoparticles have been used in antioxidant drug delivery as anti-cancerous agents by offering fast uptake and long retention time in tumor tissue after administration [121]. For example, gelatin nanoparticles have been used as a vehicle to deliver different antioxidants, such as epigallocatechin gallate, tannic acid, curcumin, and theaflavin [122].

#### 5.3.3. Other Protein-Based Nanoparticles

Silk proteins are another protein-based natural biomaterials, having unique structural properties, self-assembling ability, mechanical strength, processing flexibility, biocompatibility, and biodegradability, and therefore can be used in bioactive delivery [123,124]. On the Soy protein is another plant-based protein that has been used to produce drug nano carriers. This is one of the most common plant proteins for bioactive encapsulation. Curcumin was encapsulated in soy protein nanoparticles, with particle size ranging from 220 to 860 nm with high encapsulation efficiency of 97.2% [125]. Milk proteins such as beta-lactoglobulin and casein have been used to produce carriers for bioactives. Beta-lactoglobulin has a high potential to be used for drug delivery purposes due to its ability to retain its conformation in acidic conditions, good gelling property, and low cost [126]. Beta-lactoglobulin nanoparticles were used to preserve antioxidant activity of catechin-type natural antioxidant [127]. Casein is another milk protein with the potential for drug delivery. Casein micelles are aggregates of tens to hundreds of casein molecules that form miscellaneous structures of 100 to 200 nm. Casein has a flexible three-dimensional structure with flexibility to adopt conformational changes in various environmental conditions such as pH, ionic strength, and water activity [126]. Stability to heat and mechanical forces are among other desired properties of casein to develop nanoparticles with controlled release features [128]. Self-assembling property of casein has been used to generate nanoparticles to deliver curcumin antioxidant to cancer cell lines [129]. In another study, high antioxidant activity and cell proliferation were reported for curcumin-loaded casein nanoparticles [130]. These studies demonstrate the capability of casein micelles as nanovehicles for various lipophilic bioactive compounds, including different antioxidants such as alpha tocopherol (vitamin E).

### 5.4. Calcium Phosphate Nanoparticle

**Figure 9:**
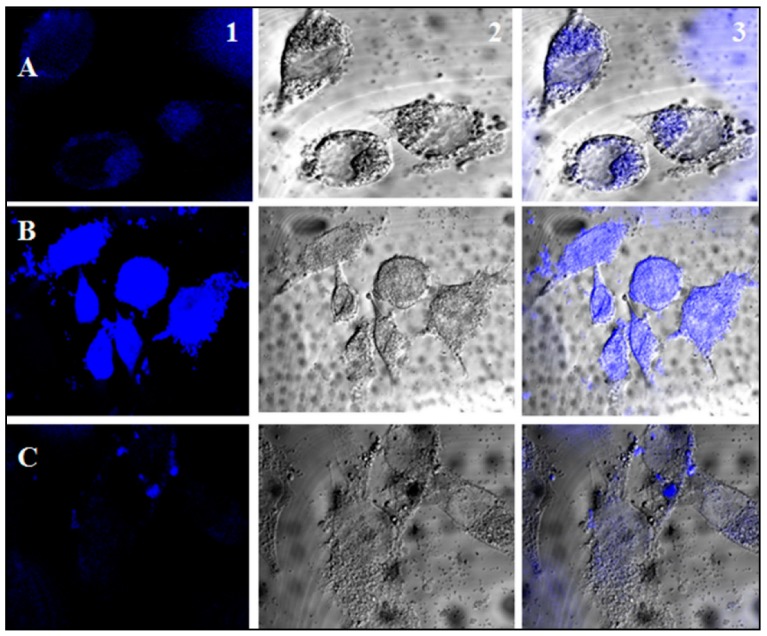
Confocal microscopic images of the CPQN- and quercetin-treated N2A cells. Cells treated with 5 μg/mL CPQN for 1 h (**A**), for 24 h (**B**), and with 5 μg/mL bulk quercetin for 24 h (**C**); 1, 2, and 3 represent blue-filtered, bright field, and overlay images, respectively. Adapted with permission from Ref. [131]. Copyright Elsevier, 2017.

Calcium phosphate nanoparticle (CPNP) is highly permeable to membrane, biocompatible, and non-toxic to the human body. In one experiment, quercetin nano-formulations were entrapped in calcium phosphate nanoparticle (CPNP) by a precipitation method at 80 °C to fabricate calcium phosphate quercetin nanocomposite (CPQN). However, loading of precursor quercetin within CPNP is pH dependent, and the highest loading percentage (83.5 ± 1.5) was found at pH 7.5, whereas the release profile of quercetin from CPQN in PBS showed an initial fast release of about 57% within the first 8 h of dialysis, followed by a slow and sustained release of the remaining quercetin (up to about 94%) in the following period, reaching a plateau after 48 h. The initial rapid release implied that over 50% of the entrapped quercetin was just adsorbed on the surface of CPNP, whereas the following sustained release signified that more than 40% of the entrapped quercetin was inside the CPNP. Moreover, interaction of quercetin with CPNP, through Ca2+ ions, enhanced its intrinsic fluorescence by more than 5000 times and thereby CPQN might be used in biological field as a potent fluorophore, having no cytotoxicity because of the high biocompatibility of the two main components of the NPs – quercetin and calcium phosphate (Figure 9). The nanocomposite had potential anti-oxidant property, because complexation with metal ions decreased the oxidation potential of flavonoids, for which mortality of mouse neuroblastoma cell N2A, by H_2_O_2_-induced oxidative stress, was found to be lowered by the pre-treatment of the cells with CPQN [131].

## 6. Conclusions and Future Perspectives

For a long time, oxidative stress has been linked to different diseases and attempted to be subsided using different medications. Conventional antioxidant therapies have been used for a long time but unfortunately have proved less effective for many reasons, including their inability to cross the blood–brain barrier, which in turn has rendered them ineffective in many neurodegenerative diseases [35]. Natural and synthetic antioxidants are now back-dated technologies for the management of oxidative stress-induced diseases. During the last few decades, inorganic nanoparticles have been successfully evaluated for their antioxidant properties, and most recently nanoantioxidants have shown the capability of attenuating oxidative stress with greater sensitivity, cellular antioxidant activity, least cytotoxic effects, and targeted delivery. In this context, covalent attachment or encapsulation of antioxidants with nanospheres of different origins, such as inorganic nanoparticles, metal nanoparticles, natural polymer-based nanoparticles, liposomes, protein-polysaccharide-based nanoparticles, and many more combinations have been tested, and are under consideration for various applications [31,140]. However, to obtain the highest benefits in terms of catalytic and biological activity from the nanoantioxidant composites, the nature, physicochemical properties, as well as mechanism of actions need to be well understood. Moreover, extensive toxicity evaluation must be needed specifically for the nonbiodegradable and insoluble nanoparticles before conducting any future biomedical application. Furthermore, beneficial aspects, as well as the side effects of the fabricated nanoantioxidants, should be identified and evaluated so that it could be used safely for in vivo application, specifically for long term treatment. Therefore, development of novel and efficient delivery of therapeutic nanoantioxidants, as well as design of novel antioxidant activity assays for accurate and reliable measurements, are the pre-requisite. Hence, better understanding of new molecules and well-developed nanostructures and nanotechnology, as well as their combination into a unique form with greater antioxidant properties, will shape up the future nanoantioxidant-mediated treatments.

## Figures and Tables

**Table 1:** Nanoantioxidants and their remarkable features.

Nanoparticles	Antioxidants and Functionalization Strategy	Remarkable Features	Ref.
SiO_2_NPs	GA; covalent grafting	Fast HAT reactions toward DPPH radicals	[30]
MSN	morin (2′,3,4′,5,7-pentahydroxyflavone); surface functionalization	Potent HO• scavenger and ^1^O_2_ quencher	[41]
MSN	Poly Tannic acid; crossing linking	Efficient antioxidant activity	[42]
MSN	Caffeic acid and rutin; covalent grafting	Antiradical functions, cellular toxicity alleviation and effective against oxidative stress	[43]
SiO_2_NPs	3,5-di-tert-butyl-4-hydroxybenzoic acid; grafting	Improved thermal oxidative stability of LDPE composite	[66]
MSN	Curcumin; loaded	Exhibited higher cellular uptake and inhibition of cancer cell viability	[67]
PEG coated AuNPs	Salvianic acid; Surface functionalization	Enhanced antioxidant and ROS scavenging in living cells	[46]
AuNPs	Trolox; Self-assembly	Enhanced antioxidant activity	[48]
AuNPs	3,6-dihydroxyflavone, lutein and selenium methyl selenocysteine; embedded	Enhanced antioxidant activity	[10]
AgNPs	Lignin; capped	Potent antioxidant; antifungal and antibacterial agents against human pathogens *S. aureus*, *E. coli*, and *A. niger*	[50]
Fe_2_O_3_NPs	GA; surface functionalization	Magnetically separable; greater antioxidant activity; outstanding antibacterial and antifungal activity	[27]
Fe_2_O_3_NPs	Carboxymethyl-inulin; coated	non-cytotoxic to the immortalized human cancer cell lines	[56]
Fe_2_O_3_NPs	Carbon; coated	Potential antioxidant, exhibited compatibility with the peripheral blood mononuclear cells	[55]
Fe_2_O_3_NPs	Poly GA, coated	Significantly reduce the oxidative stress; biocompatible and bioactive	[57]
Magnetic-silk core-shell nanoparticle	Curcumin, loaded	Greater cellular uptake and cytotoxicity in human breast cancer cell line	[58]
Ceria nanoparticles	Dextran coated and curcumin loaded	Anti-cancer properties	[61]
Ceria nanoparticles	Phospholipid-PEG; coated	Biocompatible; reduce oxidative stress, cytotoxicity, and effective agent for intracerebral hemorrhage patient	[68]
PLGA-PEG	Curcumin; loaded	Ensures neuroprotection in neonatal with hypoxic-ischemic encephalopathy	[69]
Ag-Se bimetal	Quercetin and GA	Antioxidant, antimicrobial and antitumor potentials	[62]

**Table 2:** Nanoparticles mediated antioxidants encapsulation and impacts.

Nanoparticle Carrier	Antioxidant	Nanoantioxidant Fabrication Method	Particle Size (nm)	Superiority	Ref.
Chitosan nanoformulations-AgNPs	Ascorbic acid, α-tochopherol, and catechol	Ionotropic gelation		Encapsulation efficiency: 76% Targeted delivery and sustained release to breast cancer cell, hemocompatible	[91]
CS-TPP stabilized nano and pickering emulsion	Curcumin	Ionic gelation	-	Radical scavenging activity	[92]
PPADT encapsulated NPCS linked Cy3 nanoparticles	Curcumin			Responsive to both oxidative stress and reduced pH in inflammatory milieu To monitor in vitro drug release behavior	[93]
Tripolyphosphate and chitosan	CH	Ionic gelation	68.76 ± 1.72	Higher and prolonged antioxidant and radical scavenging activity against (DPPH, NO, H_2_O_2_)	[94]
Chitosan	CGA	Ionic gelation	~250	Encapsulation efficiency: 59% Sustained release over a period of 100 h. Less cytotoxic	[95]
Chitosan/DNA	Astaxanthin	Chemical reaction, Vacuum-evaporation	92 ± 1	Prompt cellular uptake by Caco-2 cell Improved cellular viability and ROS scavenging activity (2 fold more than free astazanthin)	[96]
BSA	Quercetin	hydrophobic interaction	<10	Promotes stability of encapsulated quercetin while maintaining its antioxidant activity	[118]
Silk fibroin and chitosan polymer	Curcumin	capillary-microdot technique	<100	Higher efficiency against breast cancer cell potential to treat in vivo breast tumors by local, sustained, and long-term therapeutic delivery	[124]
Liposomes	Curcumin	mechanochemical method with a microfluidizer	263 ± 86.0	68.0% encapsulation efficiency Increased plasma antioxidant activity Enhanced bioavailability	[132]
Egg yolk phosphatidyl choline/dihexyl phosphate/cholesterol liposomal bilayer	Curcumin	Film evaporation method	64.24 ± 0.57 to 80.64 ± 0.84	Increase the nanocarrier stability	[79]
Soy lecithin liposome	Green tea catechin and epigallocatechin gallate (EGCG)	Water-oil-water emulsion	139 ± 4 to 173 ± 5	Encapsulation efficiency is more than 70% To make antioxidant rich functional food To protect and deliver antioxidant to gut	[133]
Liposomes with deoxycholic acid and dicetyl phosphate	Catechin ((+)-catechin, (−)-epicatechin, and (−)-EGCG)		378.2 ± 10.9	Encapsulation efficiency: 93.0 ± 0.1% Enhanced catechin delivery Limited skin disruption Good stability	[134]
Octaarginine-modified liposomes	Superoxide dismutase	Lipid film hydration method	170 ± 7	Fast cellular uptake and efficient cytosolic delivery of SOD. Increased scavenging efficiency of intracellular O_2_^−^	[135]
Eudragit E and PVA	Quercetin	Nanoprecipitation technique	<85	High encapsulation (99%) 74-fold higher drug delivery than pure drug Greater antioxidant activity	[136]
Polyvinylpyrrolidone	Curcumin	Nanoprecipitation technique	142.90 ± 3.12	Encapsulation efficiency (99.93 ± 0.01%) Enhanced antioxidant, drug release and antihepatoma activity	[137]
Gum arabic–maltodextrin	Epigallocatechin gallate	Spray drying	400	Highly efficient for encapsulation (96%) Integrity maintained with preserving antioxidant properties	[138]
Poly(ethylene glycol)-based nanogels	GA	Aqueous inverse miniemulsion using atom transfer radical polymerization	227 ± 51.78 to 573.3 ± 207.2	Encapsulation efficiency: 60–70% Guided controlled drug release, retained antioxidant property and biocompatible to HeLa cell lines.	[139]
Polyanhydride nanoparticles	Apocyanin	Anti-solvent nano-encapsulation method	324 to 346		[88]
